# A Fast Subpixel Registration Algorithm Based on Single-Step DFT Combined with Phase Correlation Constraint in Multimodality Brain Image

**DOI:** 10.1155/2020/9343461

**Published:** 2020-05-07

**Authors:** Jianguo Li, Quanhai Ma

**Affiliations:** ^1^People's Hospital of Xinjiang Uygur Autonomous Region, Xinjiang 830002, China; ^2^Xinjiang Uygur Autonomous Region Hospital of Traditional Chinese Medicine, Xinjiang 831199, China

## Abstract

Multimodality brain image registration technology is the key technology to determine the accuracy and speed of brain diagnosis and treatment. In order to achieve high-precision image registration, a fast subpixel registration algorithm based on single-step DFT combined with phase correlation constraint in multimodality brain image was proposed in this paper. Firstly, the coarse positioning at the pixel level was achieved by using the downsampling cross-correlation model, which reduced the Fourier transform dimension of the cross-correlation matrix and the multiplication of the discrete Fourier transform matrix, so as to speed up the coarse registration process. Then, the improved DFT multiplier of the matrix multiplication was used in the neighborhood of the coarse point, and the subpixel fast location was achieved by the bidirectional search strategy. Qualitative and quantitative simulation experiment results show that, compared with comparison registration algorithms, our proposed algorithm could greatly reduce space and time complexity without losing accuracy.

## 1. Introduction

**Figure 1:**
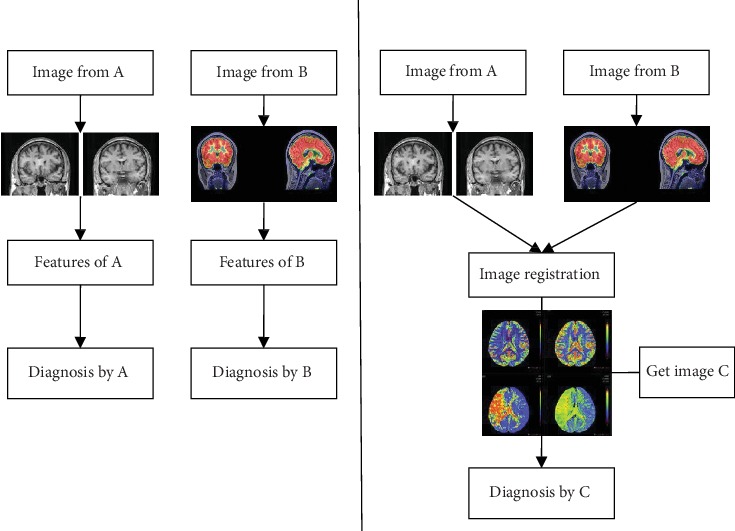
Motivation for this study.

Medical image registration technology is a widely used image processing technology in the field of medicine image analysis [1]. It plays an important role in human 3D modeling, multisource medical image fusion, the lesion feature detection and extraction, and other auxiliary diagnoses [2]. Brain medical diagnosis has a high demand for accuracy, and brain CT image registration technology is the key technology to determine the accuracy and speed of brain diagnosis and treatment [3]. The research motivation of this article is shown in Figure 1.

Different forms of images express different information and different functions. Combining the two can simultaneously express information from many aspects of the human body in one image. The internal structure and function of the human body can be reflected through the image, providing intuitive human anatomy, physiology, and pathology information. At this time, the image configuration technology needs to solve the problem of position registration of fusion between images. When there is moderate noise in the image and there is translation and scaling between the multimodality images, phase correlation image registration technology is an effective method for subpixel image registration. This paper proposes an improved algorithm based on Guizar-Sicairos registration, which can quickly search for the offset between registered images and greatly reduce the time and space complexity of registration without losing the registration accuracy.

Medical image registration can be divided into single-mode image registration and multimode image registration from the imaging mode. Single modality means that multiple images to be registered are acquired by the same imaging technology, and multimodality means that the registered images are acquired by different imaging technologies [4]. Since the imaging principles of different imaging modes are different, the images they acquire have different characteristics, and the sensitivity to different tissues is also different. Therefore, the object information of different modals is also different [5]. In order to help doctors better understand the disease situation, it is necessary to fuse a variety of multimodality images combined with more information so as to make diagnoses. For example, low-quality US images captured in real time during surgery and high-quality CT (or MRI) images before surgery are used to balance the accuracy and real time required clinically during computer-assisted intervention [6]. However, the premise of multimodal image fusion is to register multimodal images, so multimodal image registration technology is one of the research hotspots in medical image processing and is widely used in modern computer-aided interventional medicine [7]. In clinical applications, increasing clinical needs and image imaging modes have injected new impetus into multimodal medical image registration [8].

In the field of medical analysis, image registration achieves subpixel registration accuracy, but there is a huge challenge, especially in multimodality image registration [9]. In order to effectively register multimodal images, subpixel accurate feature-point location is needed. The inevitable error of the actual acquisition system makes it impossible to obtain completely accurate features of the two images, and the corresponding images are offset, stretched, and rotated in spatial domain. The research result shows that when the deviation of two complex images exceeds 0.1 pixels, the quality of the corresponding feature points will be seriously affected, which seriously affects the registration accuracy. Therefore, the high-precision registration of two multimodalities images is the first step of medical image analysis. We need to further study the key issues of multimodality registration algorithms, find the optimal algorithms that are more in line with clinical development, and further improve them reasonably.

Nowadays, typical image registration methods include registration algorithm based on control points, registration algorithm based on image features, and registration algorithm based on regional cross-correlation [10]. In particular, the registration method based on regional cross-correlation is to use cross-correlation technology to get the relative offset between image pairs, which has good robustness. Since the regional cross-correlation algorithm based on spectral operation improves the efficiency of registration, it is a common fast regional cross-correlation algorithm. Therefore, the phase correlation-based registration method has been widely studied due to its advantages of high accuracy, low computational complexity, small amount of computation, strong antinoise, and optical blur invariance [11]. The mainstream model of brain CT image registration is based on the maximum mutual information as a measurement and combined with efficient and high-precision optimization search algorithm. Because of the large amount of mutual information calculation and the slow speed of registration, the performance of the optimization search algorithm has a great impact on the efficiency and accuracy of registration. As for multimodality brain CT images, it is an effective way to improve the speed and accuracy of registration.

Phase correlation method includes spatial domain-based phase correlation and frequency domain-based phase correlation. The early correlation-registration algorithm mainly uses image translation parameters, and its image registration accuracy can achieve pixel level. Then, on this basis, Fourier–Mellin transform is used to expand image registration to the case of rotation, translation, and scaling, but its registration accuracy is also only pixel level [12]. Chen et al. proposed Fourier transform based on matrix multiplication, which can be used for subpixel registration of multimodality image [13]. Its accuracy is better than that of the traditional subpixel registration method, but the processing efficiency of large-scale multimodality medicine data is not high because the calculation efficiency of this improved method is obviously low in the process of pixel-level displacement. In order to solve the problem of mismatch (inappropriate problem) between registration accuracy and computational complexity, Claus et al. proposed an novel and effective image registration algorithm, which has the same registration accuracy as the standard fast Fourier transform and is considered to be one of the most reliable algorithms in the image registration algorithm based on the phase correlation method. For ease of description, the registration algorithm is called as SSDFT for the image registration algorithm based on the single-step discrete Fourier transform [14].

In this paper, we improve the performance of single-step discrete Fourier transform by reducing the dimension of Fourier transform cross-correlation matrix and the number of DFT matrix multiplication used to locate the peak value. Compared with other studies, the innovation of this study is summarized as follows:

1. Compared with single-step discrete Fourier transform (SSDFT), the improved algorithm proposed in this paper can quickly search the offset between the registered images
2. The method proposed enhances the registration performance of single-step discrete Fourier transform and greatly reduces the time and space complexity of registration without losing the registration accuracy
3. The algorithm proposed is robust and insensitive to noise.

The organization structure of this paper is as follows: Section 2 describes the single-step DFT registration algorithm in detail and introduces the multimodality medical image registration theory and implementation process; Section 3 proposes our improved multimodality registration algorithm, and the process of image registration is given in Section 4.  Section 5 selects the different multimodality images with manual transformation to test and verify our proposed registration algorithm and gives the simulation results and analysis; we summarize the whole paper in the last section.

## 2. Materials and Methods

### 2.1. Multimodality Brain Image Registration

Image registration refers to comparing and matching two images *F*(*x*) and *R*(*y*) obtained at different times or under different conditions. According to the spatial transformation relationship obtained from the corresponding point location information of the two images, we can define a similarity measurement function that maximizes the similarity between the two images after the spatial transformation. In other words, each point on the image *F*(*x*) has a corresponding unique point on the image *R*(*y*), and these two points should be for the same physical space location. The mathematical model of image registration is shown in the following equation:

(1) $$\begin{array}{c} S\left(T\right)=S\left(R\left(y\right),F\left(T\left(x\right)\right)\right), \end{array}$$

where *S* is the similarity measurement function; *T* is the transformation space; *R*(*y*) represents the reference image, and *F*(*T*(*x*)) represents a transformed frequency domain image. The main task of image registration is to find the optimal spatial transformation function *T* to make *S* reach the maximum, which can achieve an exact matching between the registered image and the reference image. The model is written as follows:

(2) $$\begin{array}{c} \hat{T}=\arg\underset{T}{\max}S\left(T\right), \end{array}$$

The process of registration is also the process of solving the global optimal value of the similarity measure function and its corresponding spatial transformation parameters. The search range of the parameters is called as the search space, and the number of parameters is called the degree of freedom in the spatial transformation model. The number of parameters is related to the spatial transformation model, and the degrees of freedom of different transformation models are also different [15]. Take 3D rigid body transformation as an example, spatial transformation matrix can denoted as *T*=(*t*_*x*_, *t*_*y*_, *t*_*z*_, *α*, *β*, *γ*), where *t*_*x*_, *t*_*y*_, *t*_*z*_ are the displacement offset of the registered image with respect to the three directions of the coordinate axes *x*, *y*, *z*; *α*, *β*, *γ* represent the rotation angles in three directions around the coordinate axes *x*, *y*, *z*.

**Figure 2:**
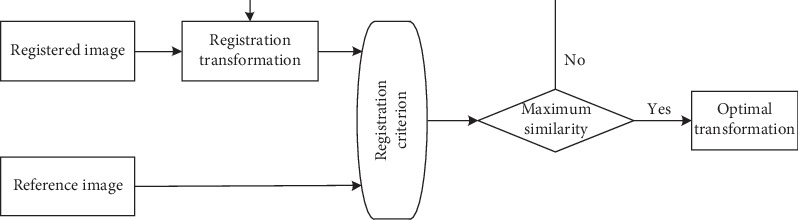
The framework of gray-based registration.

The calculation of image registration parameters can be roughly divided into two types: image registration parameters based on grayscale and image registration parameters based on features. Gray-based image registration uses the gray data for registration, which can effectively avoid errors caused by feature extraction [16]. A predefined registration measurement function in the process of medicine image registration is designed to measure the difference between two images and then search for an optimal transformation to maximize the similarity of both images. This method has the characteristics of high accuracy and strong robustness and can achieve automatic registration without preprocessing [17]. Correlation methods and mutual information methods are commonly used at present. The framework of gray-based registration is shown in Figure 2.

**Figure 3:**
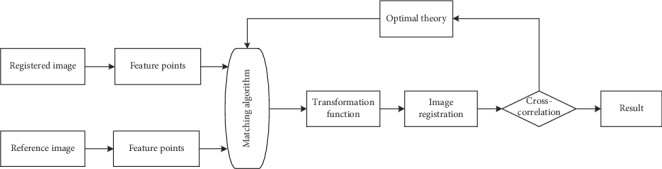
The framework of feature-based registration.

Feature-based image registration methods use feature sets extracted from images to establish a correspondence relationship between feature sets [18]. Registration parameter is the key to success, but these methods require manual participation to complete the extraction of image features. Feature-based image registration method is divided into three steps: feature extraction, feature matching, and spatial transformation. Corresponding image features are firstly extracted based on the characteristic of the image, such as corners, edges, and curvature [19]. Secondly, a matching algorithm is used to match the corresponding features between the registered image and the reference image. Finally, the best matching function of the two images is achieved by changing the transformation parameters between the registered image and the reference image. The basic steps of the feature-based image registration method are shown in Figure 3.

In feature-based image registration methods, corner points are often used to express features of medicine image. A corner point is a point where the gray level of an image changes drastically and can also be defined as the intersection of two edges in an image. Corners have the advantages of stability and abundant information. In addition, their corners also have the advantages of rotation invariance, affine invariance, and scale invariance, which is very suitable for matching medical images with different modalities [20].

### 2.2. Single-Step Discrete Fourier Transform

Correlation algorithm is a statistics-based registration [5]. It is assumed that the time interval between the two images taken successively is small enough, and there is only a small linear displacement between the images [21]; suppose that the gray distribution function of the two images is *f*_1_ and *f*_2_, and *d*_*x*_ and *d*_*y*_ are displacement offsets of *f*_2_ relative to *f*_1_ in *x* and *y* axes, respectively. The normalized mean square error (NRMSE) [22] between *f*_1_ and *f*_2_ can be expressed as follows:

(3) $$\begin{array}{c} E^{2}=\underset{\alpha ,x_{0},y_{0}}{\min}\frac{\sum_{x,y}{\left|\alpha g\left(x-x_{0},y-y_{0}\right)-f\left(x,y\right)\right|}^{2}}{\sum_{x,y}{\left|f\left(x,y\right)\right|}^{2}}\\ =1-\frac{\max\left|R_{f_{1}f_{2}}\left(x_{0},y_{0}\right)\right|}{\sum_{x,y}{\left|f_{1}\left(x,y\right)\right|}^{2}\sum_{x,y}{\left|f_{1}\left(x,y\right)\right|}^{2}}, \end{array}$$

where *r*_*f*_1_*f*_2__ represents the cross-correlation coefficient between *f*_1_ and *f*_2_. The cross-correlation value is defined as follows:

(4) $$\begin{array}{c} R_{f_{1}f_{2}}\left(d_{x},d_{y}\right)=\underset{n\times v}{\sum }f_{1}*f_{2}=\underset{n\times v}{\sum }{\hat{F}}_{1}\left(n,w\right){\hat{F}}_{2}^{*}\left(n,w\right)\\ \exp\left[i2\pi \left(\frac{d_{x}n}{M_{1}}+\frac{d_{y}w}{M_{2}}\right)\right], \end{array}$$

where ${\hat{F}}_{1}\left(\nu ,\omega \right)$ is the Fourier transform of image *f*_1_. In order to get the accurate peak position, the traditional subpixel image registration algorithm has huge storage cost and time consumption because of scaling the image and processing the whole upsampling matrix. In order to overcome this performance limitation, there are two steps proposed by Guizar-Sicairos algorithm [23] to improve the efficiency of registration: (1) the fast Fourier transform (FFT) with the upsampling coefficient *ε*_0_ = 2 is used to calculate the cross-correlation surface peak coordinates between images so as to obtain the initial subpixel motion estimation; (2) then, the accurate peak value is searched in a small window area near the initial estimation. Through the discrete Fourier transform of the small window area with the initial estimation as the center and its size as (1.5*ε*, 1.5*ε*), an upsampling cross-correlation surface is obtained, without the need of zero-padding to the product. For the realization of this process, equation (1) can also be rewritten as the matrix product of size (1.5*ε*, *M*_1_), (*M*_1_, *M*_2_), and (*M*_2_, 1.5*ε*), respectively. Therefore, the peak location can be found on the result matrix with size (1.5*ε*, 1.5*ε*). It can be seen that, compared with the conventional FFT image registration method, the computational complexity of this method is greatly improved.

## 3. The Improved Single-Step DFT for Brain Image Registration

**Figure 4:**
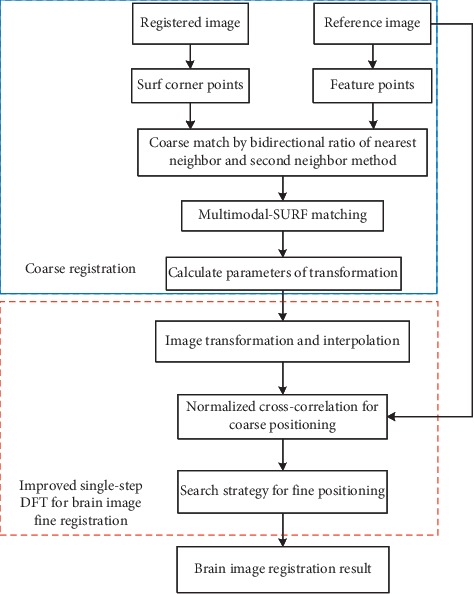
The framework of brain image registration.

Although Guizar-Sicairos algorithm is a novel and fast algorithm for subpixel registration, its main disadvantage is that most of the registration time is spent in the first step to find the initial estimation. To solve this problem, this paper improves the Guizar-Sicairos registration algorithm to reduce the time cost. Therefore, the improved algorithm is to reduce the time of initial estimation of peak position and the time of accurate registration. The framework of brain image registration is shown in Figure 4. Due to the consistency of neighborhood structures in multimodal brain images, the directions around key points where gray values change severely are used as dominant orientations. To adapt for multimodality registration, the SURF descriptor is modified according to the gradient reversals. Due to the great difference between different brain images, the existing algorithm can achieve pixel-level registration. For example, literature [8] makes full use of the flexibility of NSCT for image decomposition and the accuracy of SURF for feature location, as well as the quickness of SURF for feature extraction. The main work of this paper is to design a novel and fast subpixel large-scale translation image registration algorithm. Therefore, rough registration is represented by a blue box, and our proposed method is located in a red box.

Assuming that *F*_1_(*u*, *v*) and *F*_2_(*u*, *v*) are corresponding Fourier transforms, the following equation can be obtained:

(5) $$\begin{array}{c} F_{2}\left(u,v\right)=e^{-j2\pi \left(ux_{0}+vy_{0}\right)}F_{1}\left(u,v\right). \end{array}$$

The cross-power spectrum of two images is defined as follows:

(6) $$\begin{array}{c} P\left(u,v\right)=\frac{F_{1}\left(u,v\right)F_{2}^{*}\left(u,v\right)}{\left|F_{1}\left(u,v\right)F_{2}^{*}\left(u,v\right)\right|}=e^{j2\pi \left(ux_{0}+vy_{0}\right)}. \end{array}$$

The translation theorem of Fourier transform ensures that the phase of cross-power spectrum between images is equal to the phase difference between images. If the inverse discrete Fourier transform is applied to *P*(*u*, *v*) in frequency domain, then the unit pulse function will be obtained at (*x*_0_, *y*_0_):

(7) $$\begin{array}{c} F^{-1}\left(e^{j2\pi \left(ux_{0}+vy_{0}\right)}\right)=\delta \left(x_{0},y_{0}\right). \end{array}$$

It can be seen that the amplitude of the transformed surface is almost zero except for the amplitude at (*x*_0_, *y*_0_), so it can be used to measure the translation between two images. Since the normalization is adopted in formula (7), the phase correlation has excellent anti-interference characteristics. After the above analysis, the right side of formula (4) can be rewritten as follows:

(8) $$\begin{array}{c} R_{f_{1}f_{2}}=\underset{v,\omega }{\sum }\underset{n,m}{\sum }{\hat{F}}_{1}\left(v,\omega \right){\hat{F}}_{2}^{*}\left(v,\omega \right)\delta \left(v-K_{1}m\right)\delta \left(w-K_{2}n\right)\exp\left[i2\pi \left(\frac{d_{x}v}{M_{1}}+\frac{d_{y}\omega }{M_{2}}\right)\right], \end{array}$$

where *K*_1_ and *K*_2_ are the downsampling coefficients along the *x* direction and *y* direction, respectively, and *δ* is the Kronecker impulse function. Taking advantage of the selection feature of function *δ*, thus equation (5) can be written as follows:

(9) $$\begin{array}{c} R_{f_{1}f_{2}}=\sum_{n,m}{\hat{F}}_{1}\left(K_{1}m,K_{2}n\right){\hat{F}}_{2}^{*}\left(K_{1}m,K_{2}n\right)\exp\left[i2\pi \left(\frac{d_{x}v}{M_{1}/K_{1}}+\frac{d_{y}\omega }{M_{2}/K_{2}}\right)\right]. \end{array}$$

It can be seen that the equation represents the cross-correlation of the images after dimension reduction and sampling, where the size of the cross-correlation matrix before and after sampling is *M*_1_ × *M*_2_ and *M*_1_/*K*_1_ × *M*_2_/*K*_2_, respectively. By comparing equation (4) and equation (6), it can be inferred that as long as the peak value falls within the downsampling cross-correlation matrix, the peak positions in the matrix are the same. Therefore, the key idea of the improved algorithm in this paper is to do downsampling of the Fourier transform for the two registered images and then use the same Guizar-Sicairos method to find the initial estimation of the peak position. The complexity of the time complexity of the improved registration algorithm is *O*(*K*_1_^−1^*K*_2_^−1^*M*_1_*M*_2_*ε*).

In order to obtain accurate reconstruction based on downsampling in frequency domain, the proposed registration algorithm in this paper needs to calculate an overlapping form of cross-correlation. Because the cross-power spectrum performs *K* downsampling in two directions and the cross-correlation time spectrum is located near *M*/*K* in spatial domain, the overlap of cross-correlation function may change the initial peak position, resulting in the wrong estimation of the downsampling matrix. In order to solve this problem, the improved algorithm in this paper normalizes the original cross-correlation function before downsampling, and its expression can be denoted as follows [8]:

(10) $$\begin{array}{c} R_{f_{1}f_{2}}\left(d_{x},d_{y}\right)=e^{i2\pi \left(\nu d_{x}/M_{1}+\omega d_{y}/M_{2}\right)}. \end{array}$$

It can be seen that the inverse Fourier transform of the complex exponential is a delta function, which is to say that the position of the single peak is related to the offset (*d*_*x*_, *d*_*y*_). In order to reduce the edge effect, a window function can be used in the Fourier transform to intercept, or zero-padding. Therefore, the peak curve obtained by the cross-correlation function is independent of the image. As long as the peak position in the original normalized cross-correlation before downsampling is less than *M*/*K*, the overlapping cross-correlation will not change the initial peak position. Experimental results show that the cross-correlation surface has an obvious peak value, but the peak value surface is very broad, not much larger than other peaks. The cross-correlation surface has a sharp peak value. It means these local peaks may be considered as the position of global peaks in the wrong position area. Therefore, using the inverse Fourier transform of normalized cross-power spectrum, the matching performance will be very accurate and stable even in the presence of noise.

In order to correctly locate the peak value in the overlapping regularization phase correlation matrix, it is assumed that the subpixel translation boundaries along the *x*-axis and *y*-axis are *d*_*x*_=*M*_1_/*K*_1_ and *d*_*y*_=*M*_2_/*K*_2_, respectively. If the sampling coefficient is 2, it can be obtained the maximum subpixel displacement *d*_*x*_=*M*_1_/2 and *d*_*y*_=*M*_2_/2 in two directions. In addition, the upper limits of the sampling coefficient are *M*_1_/2 and *M*_2_/2, which will make the subpixel displacement less than 2. If these upper limits are not satisfied, the initial peak value will fall outside the boundary *M*_1_/*K*_1_ × *M*_2_/*K*_2_ of the original cross-power spectrum. It will make the peak value near these values and mistake the surface peak position, so this method cannot determine the subpixel displacement robustly.

Through the above theoretical analysis, it can be seen that the improved algorithm proposed in this paper mainly uses the downsampling cross-correlation function to solve the overlapping model and reduces the Fourier transform dimension of the cross-correlation matrix and the multiplication number of the discrete Fourier transform matrix to speed up the registration process.

## 4. Brain Image Fine-Search Strategy for Registration

The second step of Guizar-Sicairos method is to search for an accurate peak value in a small window area near the initial estimate. Combined with the above analysis, we can achieve the goal of acceleration by reducing the number of multiplication and addition of complex matrix (cumulative multiplication) needed to locate the peak value in the upsampling cross-correlation window. In addition, according to the derivation of literature [14], the correlation function can be written as the product of three matrices, namely:

(11) $$\begin{array}{c} R_{f_{2}f_{1}}={\underset{\varpi }{\sum }\left[e^{i2\pi \left(d_{y}\omega /M_{2}\right)}\right]}_{1.5\varepsilon \times M_{1}}{\underset{\varpi }{\sum }\left[{\hat{F}}_{1}\left(v,\omega \right)\times {\hat{F}}_{2}^{*}\left(v,\omega \right)\right]}_{M_{1}\times M_{2}}{\underset{v}{\sum }\left[e^{i2\pi \left(d_{x}v/M_{1}\right)}\right]}_{M_{2}\times 1.5\varepsilon }\\ =A_{1.5\varepsilon \times M_{1}}B_{M_{1}\times M_{2}}C_{M_{2}\times 1.5\varepsilon }. \end{array}$$

The aim is to find the subpixel peak in the result matrix *R*_*f*_1_*f*_2__. Through the correlation surface analysis, it can be seen that there is a correlation peak in the cross-correlation, and its upsampling form is similar to a parabola shape, and the contour is monotonically increasing. Therefore, the improved algorithm adopts a forward and backward search strategy to reduce the total number of multiplication and addition operations of complex matrix, and its search process is referred to literature [4].

The main steps of this improved algorithm are as follows: (1) coarse positioning: the algorithm uses the improved strategy proposed in Section 3 to calculate the peak coordinates of cross-correlation surfaces between images and obtain the pixel-level translation (*x*, *y*); (2) fine positioning: the DFT of improved matrix multiplication and search strategy are used to obtain the *n*-times sampled and neighborhood area 1.5 × 1.5 of the coarse positioning point (*x*, *y*), and the pixel-level translation $\left(\bar{x},\bar{y}\right)$ is obtained by calculating the phase correlation of the upsampling area. Considering the resampling multiple *n*, the subpixel-level translation $\left(\bar{x}/n,\bar{y}/n\right)$ is obtained. Therefore, the translation Δ=(*x*_1_, *y*_1_) combined with phase correlation and resampled image registration is written as follows:

(12) $$\begin{array}{c} \left\{\begin{array}{c} x_{1}=x+\frac{\bar{x}}{n},\\ y_{1}=y+\frac{\bar{y}}{n.} \end{array}\right. \end{array}$$

In this paper, phase correlation is used to obtain coarse position points in the original image and fine position point is obtained after resampling *n*-times. Because the phase correlation-based coarse positioning has the pixel-level accuracy, setting the fine positioning area as the size of 1.5 × 1.5 with the coarse positioning point as the center can ensure the subpixel-level accurate positioning point is in this area. In order to obtain high positioning accuracy and not increase too much calculation cost, the inverse Fourier transform will be carried out in the region with the size of 150 × 150 to obtain the fine positioning peak value if the upsampling times *n* is taken as 100. In other words, the positioning accuracy will reach 0.001 pixels.

It can be seen from the theoretical analysis that the improved fine-searching process in this paper will also reduce the calculation time for large-scale multimode brain imaging registration. However, both the Guizar-Sicairos algorithm and its improved algorithms use the same inverse DFT matrix, and most of the calculation time is spent on the generation of the matrix, rather than looking for the peak value. Therefore, the proposed improvement algorithm is limited to large-scale multimode brain imaging registration, but its calculation time will not exceed the original registration algorithm.

## 5. Simulation Experiment and Results

### 5.1. Experimental Data

**Figure 5:**
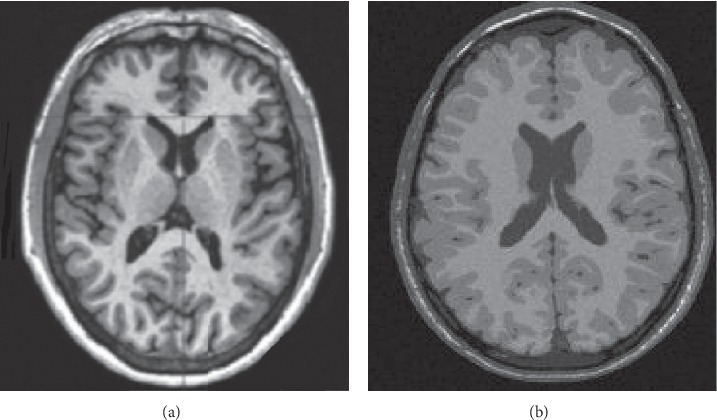
Image sample for different modalities.

This experiment uses the brain PET/CT data provided by the Second Affiliated Hospital of China Medical University (CMU) to experimentally test our proposed registration algorithm. The data obtained by PET/CT imaging equipment include a PET image volume data and a CT image volume data. The specific information of the experimental data is shown in Table 1. In addition, this experiment also applies the registration algorithm to the brain data from Vanderbilt University and will give the registration result so as to verify the accuracy of the proposed algorithm. In order to verify the registration accuracy of multimodal data acquired in the real environment, the CT brain image data acquired from a patient in Guangzhou General Military Hospital (GMH) are also selected, and the registration results are given, where the image sample is shown in Figure 5. The resolution, pixel size, and grayscale range of each image data are shown in Table 1.

### 5.2. Evaluation Index

In order to measure the registration accuracy well in the experiment, two completely aligned different modal images are selected. We adopted a random deformation operation to one image and then take the deformed image as a floating image (registered image), and the other is a reference image. The introduced random deformation operation is the gold standard to test whether the deformation obtained by registration is accurate. We measure the accuracy of image registration by calculating the difference between the gold standard and the registration estimation. In our experiment, the target registration error (THE) is the valuation index, and its definition is as follows:

(13) $$\begin{array}{c} \text{THE}=\sqrt{\frac{1}{\left|\Omega_{I}\right|}\sum_{x_{i}\in \Omega_{I}}{\left(I_{1}\left(x_{i}\right)-I_{2}\left(x_{i}\right)\right)}^{2}}, \end{array}$$

where *I*_1_ and *I*_2_ are two images that need to calculate THE; *I*_1_(*x*) and *I*_2_(*x*) are the gray values of the same points in two images; *Ω*_*I*_ is the image domain of *I*; and |*Ω*_*I*_| is the number of pixels. THE measures the similarity of the two images by calculating the root mean square error of gray value. The smaller the THE value of the reference image and the registered image is, the closer the gray value of the same point is in the two images, the more similar the whole image is, and the better the registration effect is.

### 5.3. Experiment and Results

**Figure 6:**
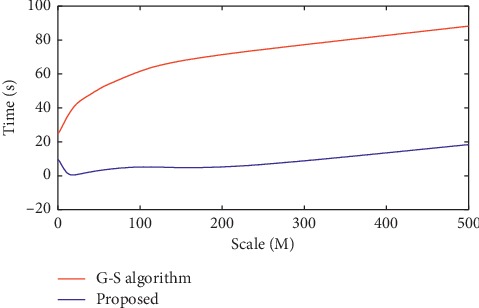
Running time for initial estimation.

**Figure 7:**
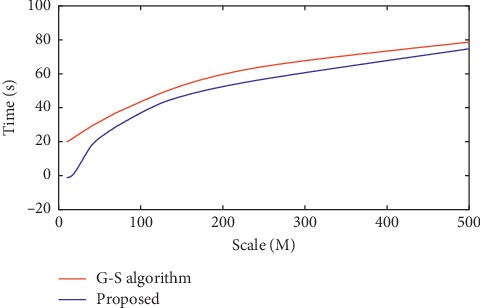
Running time for fine registration.

In order to verify the effectiveness of our proposed registration algorithm for multimodality brain images, this paper performs experimental simulation on MATLAB 7.6 platform. To fairly test the performance of the improved image registration algorithm in this paper, the Guizar-Sicairos algorithm [23], the RSTT registration algorithm proposed by Foroosh and Balci [21], the normalized cross-correlation algorithm (NCC), and the Fast-RST methods proposed by Zhou et al. [24] were selected. This paper uses the medicine images with different sizes as simulation images, and images with different scales are implemented by interpolation. In this paper, the registration accuracy of the Guizar-Sicairos method and the improved algorithm proposed in this paper are both 0.01 pixels. The calculation time required for the initial coarse peak estimation and fine-search step is shown in Figures 6 and 7. It can be seen that compared with the Guizar-Sicairos method, our proposed registration algorithm in this paper greatly reduces the time to obtain the initial peak position.

**Figure 8:**
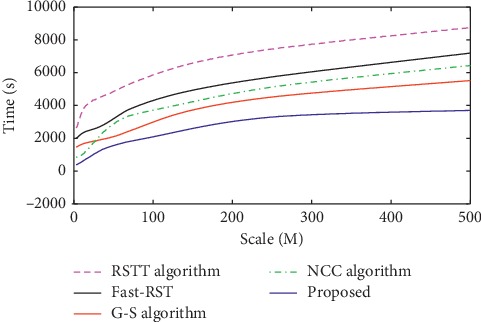
Comparison of total time for different algorithms.

For images with different sizes, the sampling coefficient is set to *M*/32. If the image size is not considered, the time required for peak estimation is roughly the same as the time required for a matrix of size 32 × 32. For large-size images with a size of 512 × 512, the estimated time is close to 2 milliseconds, while the traditional Guizar-Sicairos method requires about 200 seconds. During the fine registration process, our improved method in this paper improves the performance of the Guizar-Sicairos algorithm, as shown in Figure 7. In the entire simulation experiment, the step size *σ* is set to 0.3*ε*, and the performance comparison results of all algorithms are shown in Figure 8.

**Figure 9:**
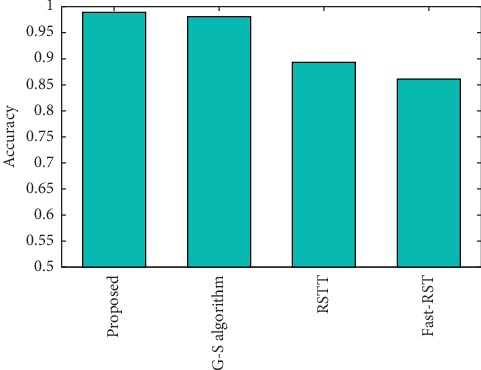
Schematic comparison of registration accuracy of each algorithm.

**Figure 10:**
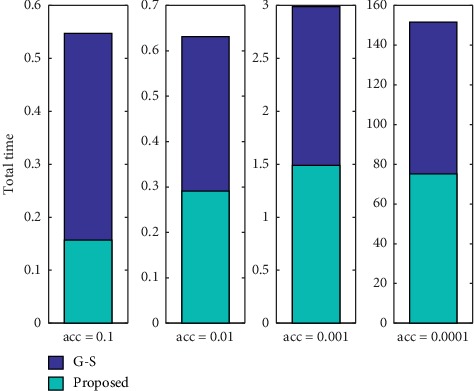
Schematic comparison of the total running time of each algorithm and registration accuracy.

Overall, the registration time of our proposed registration method is faster than that of other comparison methods. In addition, it can be seen from the experimental results that the registration error of the Guizar-Sicairos method is 0.000471, and the error of our improved method in this paper is 0.000460. The experimental process also shows that our algorithm in this paper can adapt to various translation situations. It only needs to adaptively calculate the sampling step in the initial positioning step, while the improved algorithm in [3] can only process weak translation. In order to quantitatively evaluate the accuracy of the registration algorithm, some evaluation criterion is designed for quantitative analysis.  Table 2 and Figure 9 show the effectiveness under different registration algorithms. According to the definition of target registration error (THE), if the evaluation index is close to 1, this registration algorithm is the most accurate [20]. In addition, by changing the required subpixel accuracy, this paper also tests the registration accuracy and calculation time of different comparison algorithm, as shown in Table 3 and Figure 10, where the image size is 512 × 512. Experimental results show that our improved algorithm in this paper will greatly reduce the time and space complexity for multimodality brain image registration and obtain the same subpixel accuracy as the original Guizar-Sicairos algorithm.

### 5.4. Discussion

Although Guizar-Sicairos algorithm is a novel and fast algorithm for subpixel registration, its main disadvantage is that most of the registration time is spent in the first step to find the initial estimation. To solve this problem, this paper improves the Guizar-Sicairos registration algorithm to reduce the time cost. Therefore, our improved algorithm is to reduce the time of initial estimation of peak position and the time of accurate registration. Due to the consistency of neighborhood structures in multimodal brain images, the directions around key points where gray values change severely are used as dominant orientations. To adapt for multimodality registration, the SURF descriptor is modified according to the gradient reversals. Due to the great difference between different brain images, the existing algorithm can achieve pixel-level registration. For example, literature [8] makes full use of the flexibility of NSCT for image decomposition and the accuracy of SURF for feature location, as well as the quickness of SURF for feature extraction.

Multimodality brain image registration technology is the key technology to determine the accuracy and speed of brain diagnosis and treatment. In order to achieve high-precision image registration, a novel and fast subpixel large-scale translation image registration algorithm was proposed. Firstly, the coarse positioning at the pixel level was achieved by using the downsampling cross-correlation model, which reduced the Fourier transform dimension of the cross-correlation matrix and the multiplication of the discrete Fourier transform matrix, so as to speed up the coarse registration process. Then, the improved DFT multiplier of the matrix multiplication was used in the neighborhood of the coarse point, and the subpixel fast location was achieved by the bidirectional search strategy. Simulation experiment results show that, compared with common image registration algorithms, our proposed algorithm could greatly reduce space and time complexity without losing accuracy.

## 6. Conclusion

When there is moderate noise in the image and there is translation and scaling between the multimodality images, phase correlation image registration technology is an effective method for subpixel image registration. This paper proposes an improved algorithm based on Guizar-Sicairos registration, which can quickly search for the offset between registered images and greatly reduce the time and space complexity of registration without losing the registration accuracy. Theoretical analysis and experimental verification show that our proposed multimodality brain image registration algorithm has high matching accuracy and antinoise performance, can be well applied to medicine image registration with big-scale translation, and is suitable for medicine analysis engineering applications.

## Figures and Tables

**Table 1:** Image characteristic for different modalities.

Data sources	Modality	Resolution	Pixel size	Grayscale
Vanderbilt	CT	512 × 512 × 15	0.415/0.415/6	0∼4096
	MRI	512 × 416 × 19	0.4462/0.4492/5	0∼1149

CMU	CT	512 × 512 × 28	0.6535/0.6535/4	−32,768∼32767
	PET	128 ∗ 128 ∗ 16	2.59072/2.59072/8	−32,768∼32767

GMH	CT	512 × 512 × 15	0.4151/0.4151/6	0∼4096
	MRI	512 × 416 × 19	0.4462/0.4492/5	0∼1149

**Table 2:** Comparison of registration accuracy for different algorithms.

Proposed	G-S algorithm	RSTT	Fast-RST	NCC
0.996	0.989	0.981	0.893	0.861

**Table 3:** Comparison results between total time and registration accuracy for different algorithms.

Accuracy	G-S algorithm	Proposed
0.1	0.547	0.157
0.01	0.631	0.291
0.001	2.989	1.489
0.0001	151.560	75.130

## Data Availability

All relevant data are within the paper.
